# New and Old German Bridge-Work

**Published:** 1890-08

**Authors:** L. C. Bryan

**Affiliations:** Basel, Switzerland


### NEW AND OLD GERMAN BRIDGE-WORK.

BY L. C. BRYAN, D.D.S., BASEL, SWITZERLAND.

During a recent visit to Germany, in which I visited a number of native, and resident American, dentists, I was much interested by a visit to Herr C. Rauhe, of Düsseldorf, on the Rhine. At the entrance of an elegant building on one of the principal streets of the city appears the sign "C. Rauhe, Zahntechnicker" (mechanical dentist). This means that the dentist has not gone through the regular stages of dental education, and passed the examination entitling him to the use of the word Zahnarzt (literally, tooth doctor), although his practice is identical with that of the Zahnarzt.

In the L of the house, on the first floor of which the dentist lives and has his practice, is the factory, which has developed in a few years from a dental laboratory, and from which he supplies a large part of the European dentists with all kinds of engine fixtures, from drills and burs to straight and right-angle handpieces, pneumatic hammers of various modifications, with direct, right-, and obtuse-angle blows, besides various other useful dental contrivances, largely of his own invention, or copies of American manufacture.

But what interested me the most during my visit, and that to which I desire to give publicity, is a novel construction of removable bridge-work, which he patented in May of 1879. This patent was taken out for a year, simply to record it as Mr. Rauhe's invention, and has never been renewed, and is now public property. During my visit, a patient called "to have her bridge tightened," and I had an opportunity of examining the construction of a full upper set without palate plate, the narrow rim of which fitted the ridge of the alveolus, and was secured in place by pins anchored with amalgam in three incisor and cuspid roots. The patient remarked that the set had done good service, and given satisfaction for several years. The pins extending out of the roots were originally split and spread apart to spring into place and to secure a firm hold in corresponding holes in the plate; but Mr. Rauhe now makes them solid, and inserts in the plate-holes gold spiral springs similar to the springs still largely used in Europe to retain ill-fitting double sets in the mouth. When, from wear, the bridge-work loosens, this spiral spring is narrowed in its diameter by pressing an excavator between it and the plate, or, with narrow-beaked pliers, bent inward.

The patient removes the bridge for cleaning, and replaces it with apparent ease. It is made of rubber, strengthened, if desired, with gold or other wire. It is as clean as any fixture which rests on roots, and struck me as very ingenious and practical. The pins are anchored in the roots, and no matter what the various angles of the inclination of the roots, the pins can all stand perfectly perpendicular and parallel above the end of the root, and so offer no difficulty to the insertion of the plate; while pins attached to a plate or bridge would have to stand at the various angles of the canals of the roots to which they are adjusted, thus precluding the use of a bridge with pivots in some cases. Another advantage of securing the pins in the root is the preservation of the root from decay, and from wear.

In anchoring the pin, the filling, either of amalgam or of gold, should entirely cover the end of the root, thus preserving it indefinitely from decay.

The principle of securing a bridge or bar of gold with teeth attached, by means of pins running into roots of teeth, is very old in Germany and France; and I send you herewith a bridge which is fifty years old, and was worn by the mother of an old lady patient of mine. The teeth, mounted by a village dentist, are probably of French make, and quite different from those now manufactured, and are without natural form or appearance, having a porcelain body with thin enamel surface, and three very short pins on the borders of a groove at the back. In this groove a gold pin was laid, instead of a plate backing. The pins were bent over or against it, and soldered. The teeth so backed were soldered to a strip of gold which was in turn provided with pivots to secure it to the roots, cotton fibres or floss silk being wound round the pins to secure them in the roots. The four platina-backed incisors, with two pivots extending into the central incisor roots, were made eighteen years ago by my able Basel colleague, Dr. August Ritmann, and held firmly with their cotton fibre fastenings until all the remaining teeth had decayed, and were recently removed to make room for a full upper set.

